# Associations of Smoking With Delirium and Opioid Use in Older Adults With Traumatic Hip Fracture

**DOI:** 10.5435/JAAOSGlobal-D-21-00188

**Published:** 2022-05-13

**Authors:** Kristin Salottolo, Richard Meinig, Landon Fine, Michael Kelly, Robert Madayag, Francie Ekengren, Allen Tanner, David Bar-Or

**Affiliations:** From the Trauma Research Department, Penrose Hospital, Colorado Springs, CO (Salottolo, Dr. Meinig, Kelly, Dr. Tanne II, and Dr. Bar-Or); the Trauma Research Department, St Anthony Hospital, Lakewood, CO (Salottolo, Dr. Madayag, and Dr. Bar-Or); the Trauma Research Department, Wesley Medical Center, Wichita, KS (Salottolo, Dr. Ekengren, and Dr. Bar-Or); the Trauma Research Department, St. Francis Medical Center, Colorado Springs, CO (Dr. Meinig); and the Trauma Research Department, Parker Adventist Hospital, Parker, CO (Dr. Fine).

## Abstract

**Introduction:**

Cigarette smoking is a risk factor for hip fractures, while risk factors for developing delirium include older age and preexisting cognitive impairment. We sought to determine whether smoking status is independently associated with delirium and pain outcomes.

**Methods:**

This was a prospective, observational cohort study of 442 older adults (65 to 90 years) admitted for traumatic hip fracture at five trauma centers. The primary exposure was smoking status (n = 43, 10%). Additional risk factors included demographics, injury characteristics, and medical interventions. Delirium (primary) and analgesia-related complications were examined with multivariable logistic regression, while analysis of covariance models were used to examine preoperative and postoperative pain scores and opioid consumption (oral morphine equivalents).

**Results:**

Smokers had significantly worse outcomes compared with nonsmokers: delirium incidence was 16.3% versus 5.0% (adjusted odds ratio, 4.23; *P* = 0.005), analgesia complications developed in 30.2% versus 14.8% (adjusted odds ratio, 2.63; *P* = 0.01), and postoperative opioid consumption was greater (53 mg versus 33 mg, adjusted *P* = 0.04). Adjusted pain scores were not different between groups.

**Discussion:**

Smoking status is associated with markedly worse outcomes in older adults with traumatic hip fracture. Smoking status should be considered in pain management protocols and for early screening and delirium prevention methods.

**Data availability:**

On reasonable request.

Complications after surgical management of hip fractures are common and occur in 75% of patients.^1^ The most common complication is delirium, which ranges from 4% to 53%.^2^ Delirium is associated with notable morbidity and mortality including longer length of stay (LOS), more complications, worse functional status, and increased mortality.^3-5^ In older adult patients with hip fracture, current risk factors for developing delirium have been extensively researched and most commonly include older age and preexisting cognitive impairment (eg, dementia).

In patients undergoing elective knee or hip arthroplasty, there is no association between smoking status and delirium. A study of nearly 42,000 patients having elective procedures reported smoking in 25% of patients with delirium and 24% without delirium.^6^ A meta-analysis evaluating 28 potential risk factors for postoperative delirium in patients with hip or knee arthroplasty did not find smoking to be associated.^7^

Patients having elective hip arthroplasty differ from patients with acute, traumatic hip fractures needing repair or arthroplasty. The latter group of patients are older, have more medical comorbidities, longer lengths of stay, higher intensive care unit (ICU) admission and readmission rates, more postoperative complications, higher mortality, and higher costs of care than patients having elective procedures.^8,9^ In populations that include traumatic hip fracture, the association between delirium and smoking is less clear, potentially because of case mix, varying definitions of delirium, or confounders evaluated in the adjusted models.^10-12^

Given the disparate findings in the literature, the purpose of this prospective multicenter cohort study was to determine whether smoking status is independently associated with delirium and other poor outcomes in older adult patients with acute, traumatic hip fracture.

## Methods

This study was conducted by the Injury Outcomes Network, a collaborative research network of community-based level I and II trauma centers. The following hospitals enrolled patients: Penrose-St. Francis Hospital, Colorado Springs, CO; St. Anthony Hospital, Lakewood, CO; Parker Hospital, Parker, CO; and Wesley Medical Center, Wichita, KS. This study received approval from the HealthONE Institutional Review Board and required documentation of informed consent and from the Catholic Health Initiatives Institutional Review Board with a waiver of informed consent.

Patients were prospectively screened daily by dedicated trauma clinical research coordinators from January 2019 to November 2020 for the following inclusion criteria: aged 65 to 90 years, a traumatic hip fracture requiring surgery, and arrival within 12 hours of injury. Patients were excluded based on notable multiple trauma (defined by injury severity score [ISS] > 16), coagulopathy (defined by INR > 1.8 or administration of agents or blood products intended for anticoagulant reversal), bilateral hip fractures, lack of documented confusion assessment method (CAM) assessments in the preoperative and postoperative periods, and preexisting cognitive impairment (eg, dementia or Alzheimer disease diagnosed before the acute hospitalization, as stated in the patient's medical record).

The primary exposure was smoking status, defined as current smoker and nonsmoker of any tobacco products (cigarettes, vaping, chew, and pipe); all patients were cigarette users. We also examined heavy smoking, defined by the World Health Organization as at least one pack per day. Additional risk factors included demographics (age, sex, race, and American Society of Anesthesiologists score [coded as I/II or ≥ III]), comorbidities (examined if they occurred in at least 5% of the population), ISS, cause of injury (fall or other cause), fracture type (head or neck, intertrochanteric, and subtrochanteric), and medical interventions: surgical repair or arthroplasty or percutaneous repair, general or regional anesthesia, use of fascia iliaca compartment block (FICB), and nicotine replacement therapy (NRT). NRT is offered for current smokers, although it is often refused during the hospitalization.

The primary study end point was the development of delirium within 48 hours postoperatively. Delirium was assessed by validated CAM and CAM-ICU assessment tools, which were typically used with each shift change or if there was a change in mental status. Other end points included analgesia-related complications (urinary retention, respiratory depression, hypotension, constipation, block failure, and overdose), self-reported pain numeric rating scale scores (0, no pain, to 10, worst imaginable pain) at prespecified time points but particularly in the preoperative and postoperative periods, and total opioid consumption in the preoperative and postoperative periods reported using equianalgesic conversion to oral morphine equivalents (OMEs).^13^ All end points were manually abstracted from the patient's chart by dedicated trauma research staff.

Statistical analysis was conducted with SAS (SAS Institute). There was no imputation of missing data. All covariates with *P* < 0.15 in univariate comparison with smoking status were included in the final models. The independent association of smoking status on delirium and analgesia-related complications was examined with multivariate logistic regression models. The independent association of pain scores and OMEs was examined using analysis of covariance models. OMEs were modeled with a log transformation to achieve normality. A two-sided *P* < 0.05 was considered statistically significant.

## Results

Of 442 older adult patients, 10% (n = 43) were smokers. Compared with nonsmokers, smokers were younger (median age: 72 versus 79 years, *P* < 0.001) and more likely to have chronic obstructive pulmonary disease (COPD; 35% versus 13%, *P* < 0.001), Table 1.

**Table 1:** Univariate Associations With Smoking Status

Covariate, % (n)	Smoker (n = 43)	Nonsmoker (n = 399)	*P*
Median (IQR) age, yrs	72 (68-78)	79 (73-85)	**<0.001**
Female sex	65.1% (28)	65.4% (261)	0.97
ISS > 9 (other minor injury)	32.6% (14)	28.4% (113)	0.57
White race	95.4% (41)	92.7% (370)	0.76
Fall cause of injury	97.7% (42)	96.2% (384)	1.00
ASA score ≥III	76.7% (33)	68.7% (274)	0.27
Any comorbidity	76.7% (33)	80.0% (319)	0.62
Hypertension	48.8% (21)	60.9% (243)	0.13
Diabetes	14.0% (6)	17.3% (69)	0.58
Dependent	16.3% (7)	19.3% (77)	0.63
COPD	34.9% (15)	12.5% (50)	**<0.001**
Anticoagulant	14.0% (6)	20.3% (81)	0.32
Advanced directive	11.6% (5)	12.3% (49)	0.90
Injury and treatment information			
Fracture type			0.54
Head or neck	59.5% (25)	55.1% (218)	
Intertrochanteric	40.5% (17)	42.4% (168)	
Subtrochanteric	0% (0)	2.5% (10)	
Surgical procedure			0.86
Hip arthroplasty	37.2% (16)	41.4% (165)	
Hip repair	37.2% (16)	35.6% (142)	
Hip repair, percutaneous	25.6% (11)	23.1% (92)	
General anesthesia	81.4% (35)	87.2% (348)	0.29
FICB	69.8% (30)	73.9% (295)	0.56
Median (IQR) time to FICB	5.3 (3-73)	3.7 (3-6)	0.06
Delayed surgery > 24 hr	23.3% (10)	20.6% (82)	0.68

ASA = American Society of Anesthesiologist classification, COPD = chronic obstructive pulmonary disease, FICB = fascia iliaca compartment block, ISS = injury severity score, IQR = interquartile range, SE = standard error

Data in bold denote statistical significanceat a threshold of *P* < 0.05.

Most of the smokers (57%) were considered to have heavy consumption of ≥1 pack/d. There were few demographic or clinical differences between heavy-consumption and non–heavy-consumption smokers; heavy smokers were more likely to receive NRT (85% versus 47%, *P* = 0.03) and more likely to have hip repair over arthroplasty (Table 2).

**Table 2:** Univariate Associations by Heavy Smoking Status

Covariate, % (n)	Heavy smoker^a^ (n = 20)	Regular smoker (n = 15)	*P*
Median (IQR) age, yrs	75 (68-80)	72 (67-76)	0.20
Female sex	65.0% (13)	66.7% (7)	0.92
ISS > 9 (other minor injury)	35.0% (7)	26.7% (4)	0.72
White race	95.0% (19)	93.3% (14)	1.00
Fall cause of injury	95.0% (19)	100.0%	1.00
ASA score ≥III	75.0% (15)	80.0% (12)	1.00
Any comorbidity	75.0% (15)	73.3% (11)	1.00
Injury and treatment information			
Head or neck fracture	52.6% (10)	73.3% (11)	0.22
Nicotine replacement therapy	85.0% (17)	46.7% (7)	**0.03**
Hip arthroplasty (vs. repair)	15.0% (3)	60.0% (9)	**0.02**
General anesthesia	85.0% (17)	66.7% (10)	0.25
FICB	70.0% (14)	73.3% (11)	1.00
Delayed surgery > 24 hr	20.0% (4)	26.7% (4)	0.70

ASA = American Society of Anesthesiologist classification, FICB = fascia iliaca compartment block, IQR = interquartile range, ISS = injury severity score

a Heavy smoker defined as one pack/d. Details of smoking consumption were missing in eight smokers.

Data in bold denote statistical significanceat a threshold of *P* < 0.05.

Smokers who had NRT were less likely to have ISS <9 than smokers who did not receive NRT (21% versus 47%), although this did not reach statistical significance (*P* = 0.07; Supplementary Table 1, http://links.lww.com/JG9/A217). There were no other demographic or clinical differences by treatment with NRT.

**Figure 1:**
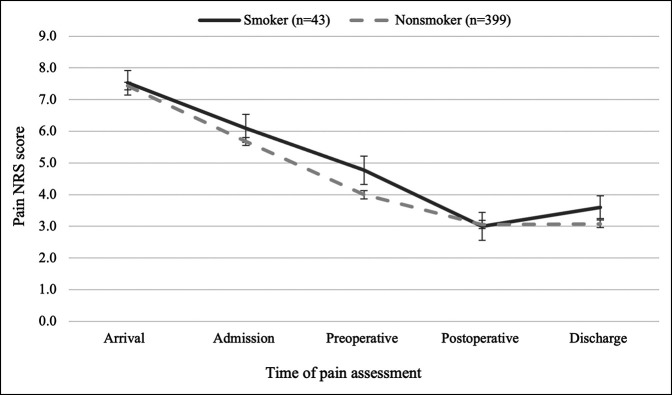
Graph demonstrating the average (standard error) pain numeric rating scale (NRS, 0-10 scale) scores at prespecified time points during the hospitalization.

Before adjustment, smokers had significantly worse outcomes compared with nonsmokers (Table 3). Delirium incidence was 16.3% in smokers versus 5.0% for nonsmokers (*P* = 0.01), analgesia complications developed in 30.2% smokers versus 14.8% nonsmokers (*P* = 0.01), and OME consumption was greater preoperatively (OMEs: 45 mg versus 30 mg nonsmokers, *P* = 0.01) and postoperatively (OMEs: 53 mg versus 33 mg nonsmokers, *P* = 0.001). As shown in Figure 1, pain scores were not different between groups at any of the prespecified time points.

**Table 3:** Unadjusted Outcomes by Smoking Status

Covariate, % (n)	Smoker (n = 43)	Nonsmoker (n = 399)	*P*	Heavy smoker^a^ (n = 20)	Regular smoker (n = 15)	*P*
Outcomes						
Delirium	16.3% (7)	5.0% (20)	**0.01**	15.0% (3)	26.7% (4)	0.43
Analgesia complication	30.2% (13)	14.8% (59)	**0.01**	30.0% (6)	46.7% (7)	0.31
OMEs, median (IQR)						
Preoperative	45 (15-81)	30 (12-53)	0.01	21 (14-58)	75 (63-102)	**0.01**
Postoperative	53 (30-168)	33 (12-75)	0.001	50 (21-111)	75 (43-168)	0.29
Pain NRS, mean (SE)						
Preoperative pain	4.8 (0.45)	4.0 (0.13)	0.10	4.2 (0.61)	4.8 (0.93)	0.59
Postoperative pain	3.0 (0.44)	3.1 (0.13)	0.89	2.4 (0.57)	3.4 (0.95)	0.37

IQR = interquartile range, OME = oral morphine equivalent, NRS = numeric rating scale score, SE = standard error.

a Heavy smoker defined as one pack/d. Details of smoking consumption were missing in eight smokers. Data in bold denote statistical significance at a threshold of *P* < 0.05.

Unadjusted outcomes for heavy-consumption versus non–heavy-consumption smokers were similar, except for OMEs (Table 3). Heavy smokers consumed fewer opioids preoperatively (21 versus 75 mg, *P* = 0.01) than nonheavy smokers. Unadjusted outcomes were also similar by NRT, except preoperative pain scores that were significantly lower in smokers receiving NRT (mean: 4.0) than smokers without NRT (mean: 5.8) (*P* = 0.04).

After adjustment for age, chronic hypertension, and COPD, smokers had significantly worse outcomes than nonsmokers (Tables 4 and 5). The odds of delirium increased fourfold for smokers compared with nonsmokers (adjusted odds ratio [AOR], 4.2, *P* = 0.005), the odds of an analgesia complication increased more than twofold for smokers compared with nonsmokers (AOR, 2.6; *P* = 0.01), and opioid consumption was greater for smokers compared with nonsmokers postoperatively (*P* = 0.04). Moreover, no other covariates were markedly associated with delirium after considering smoking status. In addition to smoking, risk factors for analgesia-related complications included chronic hypertension. In addition to smoking, risk factors for higher postoperative OME consumption included younger age and COPD. Adjusted pain scores were not different by smoking status (preoperatively: *P* = 0.39; postoperatively, *P* = 0.47); only COPD was independently associated with worse pain scores (Table 5).

**Table 4:** Multivariate Logistic Regression Analysis

Covariate	Delirium OR (95% CI)	Analgesic complication OR (95% CI)
Smoker vs. nonsmoker	4.23 (1.5-11.5)^a^	2.63 (1.2-5.6)^a^
Age 80–90 vs. 65–79 yrs	1.63 (0.7-3.7)	0.96 (0.6-1.6)
Hypertension vs. not	1.42 (0.6-3.3)	2.06 (1.2-3.6)^a^
COPD vs. not	1.23 (0.4-3.4)	1.19 (0.6-2.4)

CI = confidence interval, COPD = chronic obstructive pulmonary disease, OR = odds ratio

a Denotes statistical significance with *P* < 0.05.

Adjusted for variables with *P* < 0.15 in univariate analysis.

**Table 5:** Multivariate Linear Regression Analysis

Covariate	Preoperative OME^a^	Postoperative OME^a^	Preoperative pain scores	Postoperative pain scores
Smoker vs. nonsmoker	3.4 vs. 3.1	4.0 vs. 3.4^b^	4.8 vs. 4.5	3.2 vs. 3.5
Age 80–90 vs. 65–79yo	3.1 vs. 3.5^b^	3.5 vs. 3.9^b^	4.5 vs. 4.9	3.4 vs. 3.3
Hypertension vs. not	3.2 vs. 3.4	3.7 vs. 3.7	4.6 vs. 4.7	3.2 vs. 3.5
COPD vs. not	3.4 vs. 3.1	4.0 vs. 3.4^b^	5.3 vs. 4.0^b^	3.8 vs. 2.8^b^

COPD = chronic obstructive pulmonary disease, OME = oral morphine equivalent

a Reported with log transformation.

b Denotes statistical significance with *P* < 0.05.

Adjusted for variables with *P* < 0.15 in univariate analysis.

## Discussion

This study of older adult patients with acute, traumatic hip fracture demonstrates that smokers have markedly worse outcomes than nonsmokers, with a greater development of delirium and analgesia-related complications and greater opioid consumption. Pain scores were similar for smokers and nonsmokers despite, or because of, greater opioid consumption. We postulate that the analgesic effects of nicotine use combined with smoking cessation and nicotine withdrawal led to hyperalgesia, resulting in greater narcotic use to maintain adequate pain levels, in turn leading to a greater development of delirium and analgesia-related complications.^14-16^ A propensity-matched study examining smoking status and pain in nontraumatic total hip arthroplasty also reported increased opioid consumption for smokers without a notable increase in pain intensity.^17^

Previous studies using the American College of Surgeons National Surgical Quality Improvement Program (ACS-NSQIP) database, which includes a case mix of both elective cases and fall from standing (eg, acute, traumatic hip fracture), report disparate findings of an association between smoking and delirium. An analysis of 6,210 patients from the NSQIP database reported a lower prevalence of smoking in patients with delirium than those without delirium (8.0% versus 10.3%), but an increased risk of postoperative delirium for smokers after adjustment (AOR, 1.36; *P* = 0.01).^10^ In a second study of 8,439 patients with hip fracture in the NSQIP database, the prevalence of smoking was trending lower in patients with delirium than patients who did not develop delirium (7.4% versus 8.8%, *P* = 0.054), but there was no association after adjustment (AOR, 1.14; *P* = 0.33).^11^ In a third analysis conducted in a subset of 1,261 patients from the NSQIP database undergoing nonelective surgery for hip fracture, there was a similar prevalence of smoking in patients who developed delirium and those without delirium (9.6% versus 9.1%), yet a nearly threefold increased odds of delirium for smokers after adjustment was observed (AOR, 2.88; *P* = 0.008).^12^ Our study of exclusively traumatic hip fracture demonstrated an increased incidence of delirium among smokers, before and after adjustment.

In other settings and populations, smoking is not consistently identified as a risk factor for delirium. There was a positive association between smoking and delirium in medical/surgical ICU patients,^18^ patients with acute ischemic stroke,^19^ and in a medical ICU setting.^20^ In ventilated patients, the incidence of agitation, but not delirium, markedly increased for smokers compared with nonsmokers.^21^ Conversely, two recent systematic reviews of critically ill and ICU patients found no definitive association between smoking and delirium.^22,23^

There is more consistent evidence to support an association between smoking and greater opioid consumption with acute postoperative pain among patients with knee and hip arthroplasty,^16^ total hip arthroplasty,^17^ and major general surgery.^24^ Smokers in our study had 40% greater consumption of opioids preoperatively and 46% postoperatively compared with nonsmokers.

COPD was a notable predictor of greater postoperative OME use and pain scores. Possibly, this finding of an association between COPD and OME consumption reflects the common use of opioids in older adults with COPD, reported to be 23% to 68%.^25,26^ Our study did not find COPD to increase the odds of developing delirium or analgesia-related complications. Dubois et al.^18^ similarly identified smoking, but not COPD, increased risk for delirium, while a meta-analysis identified COPD as one of 14 risk factors for delirium, albeit a weak association (relative risk [RR], 1.08; 95% CI, 1.01 to 1.16).^27^ In our study, smokers were three times as likely to have COPD. Additional investigation should attempt to clarify independent effects of smoking and COPD on outcomes in a larger population to limit residual confounding.

Heavy-consumption smokers and non–heavy-consumption smokers were similar demographically and clinically, but heavy smokers were more likely to receive NRT and consumed fewer opioids preoperatively. However, there was no association between NRT and most outcomes. Only preoperative pain was markedly lower for smokers receiving NRT than smokers who did not. Transdermal or intranasal NRT does not consistently result in better outcomes for smokers. A Cochrane review of nine randomized controlled trials examined NRT and found a very small benefit on postoperative pain at 24 hours but not at earlier time points and no effect on opioid consumption or analgesic complications, except for a higher risk of nausea than placebo.^28^ Additional systematic reviews of the efficacy of NRT reported a reduction in cumulative opioid consumption at 24 hours but more postoperative nausea compared with control subjects^29^ and inconclusive findings for NRT on delirium management in the ICU.^30^

There are three main clinical take-home messages from our study. First, smoking status should be used for early screening and recognition of delirium, which may lead to earlier intervention such as targeted delirium prevention protocols. There is a lack of effective pharmacologic treatment options for delirium, and prevention methods are prioritized.^31,32^

Second, smokers in our study had a median OME consumption that was double that of nonsmokers in both the preoperative and postoperative periods. While smoking cessation cannot be promoted before nonelective surgical repair of acute traumatic hip fracture, healthcare providers should be cognizant of greater proximate and long-term opioid prescribing for smokers and promote smoking cessation methods. In a meta-analysis of nearly 2 million patients, tobacco use was markedly associated with prolonged use of opioid prescription filling greater than 3 months postoperatively.^33^ Specifically among patients with total knee or hip arthroplasty, smokers required 90% more morphine equivalents in the 3 months after surgery than nonsmokers.^34^ Increased opioid use for smokers persisted even with legislation meant to curb opioid prescriptions.^35^

Third, approximately 80% of patients have inadequate postoperative pain control, which is associated with worse outcomes in hospital and longer term.^36^ Smokers experiencing nicotine withdrawal have lower pain tolerance than that of nonsmokers as early as 48 hours after cessation.^37^ Our findings suggest that smokers may require more frequent pain assessments for optimal pain management because severe pain with hip fracture increases risk of delirium. Paradoxically, delirium risk is increased both with opioid use^38^ and with inadequate opioid administration.^39^

There are several study limitations. First, we excluded patients with preexisting cognitive impairment, which limits the generalizability of our findings. Baseline dementia is both highly prevalent with hip fracture (approximately 30% of patients from the American College of Surgeons-NSQIP database)^12^ and highly associated with developing delirium.^2,7,11,40^ Second, detailed smoking information, such as packs per day, was missing in 19% of the smokers (n = 8). Third, delirium was more common in smokers with NRT than those without NRT (25% versus 5%, *P* = 0.11), although this association was not statistically significant. We are unable to determine whether NRT was prescribed because of the onset of delirium or whether delirium worsened or improved with NRT.

A final limitation of this analysis was that this study was not statistically powered to examine smoking on delirium; rather, this analysis was a secondary aim of a prospective study that was powered to examine the effect of FICBs on delirium. We determined that FICBs did not influence delirium or opioid consumption, but univariately, there was an association between smoking status and delirium (submitted for publication). The present analysis identified a statistically significant association between smoking and development of complications including delirium after adjusting for clinically relevant and statistically significant differences between primary exposure (smoking) and outcome (delirium).

In conclusion, smoking is associated with markedly worse outcomes in older adults with traumatic hip fractures, including delirium, opioid consumption, and analgesic-related complications. Smoking status should be considered in pain management protocols and for early screening and delirium prevention and treatment.

## Supplementary Material

SUPPLEMENTARY MATERIAL
