# Supplementary material for: Associations of Smoking With Delirium and Opioid Use in Older Adults With Traumatic Hip Fracture
Source: J Am Acad Orthop Surg Glob Res Rev. 2022 May 13;6(5):e21.00188. doi: 10.5435/JAAOSGlobal-D-21-00188 (PMC10566843; doi:10.5435/JAAOSGlobal-D-21-00188)
Supplement: SUPPLEMENTARY MATERIAL [file jagrr-6-e21.00188-s001.docx]

| **Supplementary Table S1. Univariate associations by nicotine replacement therapy (NRT)** | | | | |
| --- | --- | --- | --- | --- |
| Covariate, % (n) | NRT (n=24) | no NRT (n=19) | p value |  |
| Median age years (IQR) | 72 (68-78) | 73 (70-81) | 0.40 |  |
| Female sex | 67% (16) | 63% (12) | 0.81 |  |
| ISS > 9 (other minor injury) | 21% (5) | 47% (9) | 0.07 |  |
| White race | 100% | 89% (17) | 0.19 |  |
| Fall cause of injury | 96% (23) | 100.0% | 1.00 |  |
| ASA score ≥III | 83% (20) | 68% (13) | 0.30 |  |
| Any comorbidity | 75% (18) | 79% (15) | 1.00 |  |
| **Injury and hospital information** | | | | |
| Head or Neck fracture | 52% (12) | 68% (13) | 0.29 |  |
| Hip replacement | 25% (6) | 53% (10) | 0.10 |  |
| General anesthesia | 83% (20) | 79% (15) | 1.00 |  |
| FICB | 71% (17) | 68% (13) | 0.86 |  |
| Delayed surgery > 24h | 25% (6) | 21% (4) | 1.00 |  |
| **Outcomes** |  |  |  |  |
| Delirium through 48h postop | 25% (6) | 5% (1) | 0.11 |  |
| Analgesia complications | 29% (7) | 32% (6) | 0.86 |  |
| Oral morphine equivalents, median (IQR) | | | | |
| preoperative | 54 (15-78) | 39 (20-90) | 0.86 |  |
| postoperative | 50 (29-111) | 60 (38-194) | 0.45 |  |
| Pain scores over time, mean (SE) |  |  |  |  |
| Arrival pain | 7.9 (0.30) | 7.1 (0.79) | 0.37 |  |
| Admission pain | 6.0 (0.61) | 6.2 (0.63) | 0.73 |  |
| Preoperative pain | 4.0 (0.60) | 5.8 (0.62) | **0.04** |  |
| Postoperative pain | 2.3 (0.53) | 3.8 (0.70) | 0.11 |  |
| Discharge pain | 3.2 (0.51) | 4.2 (0.47) | 0.16 |  |
